# Surface and build‐up dose comparison between Elekta 6 MV flattening filter and flattening‐filter‐free beams using an advanced Markus ionization chamber and a solid water‐equivalent phantom

**DOI:** 10.1002/acm2.13094

**Published:** 2020-11-12

**Authors:** Toshikazu Imae, Shigeharu Takenaka, Yuichi Watanabe, Atsushi Aoki, Kanako Matsuda, Katsutake Sasaki, Shigeki Saegusa, Kanabu Nawa, Keiichi Nakagawa, Osamu Abe

**Affiliations:** ^1^ Department of Radiology University of Tokyo Hospital Tokyo Japan; ^2^ Faculty of Health Sciences Komazawa University Tokyo Japan

**Keywords:** a solid water‐equivalent phantom, build‐up, flattening filter (FF) beam, flattening‐filter‐free (FFF) beam, ionization chamber, surface dose

## Abstract

Using a plane‐parallel advanced Markus ionization chamber and a stack of water‐equivalent solid phantom blocks, percentage surface and build‐up doses of Elekta 6 MV flattening filter (FF) and flattening‐filter‐free (FFF) beams were measured as a function of the phantom depth for field sizes ranging from 2 × 2 to 10 × 10 cm^2^. It was found that the dose difference between the FF and the FFF beams was relatively small. The maximum dose difference between the FF and the FFF beams was 4.4% at a depth of 1 mm for a field size of 2 × 2 cm^2^. The dose difference was gradually decreased while the field size was increased up to 10 × 10 cm^2^. The measured data were also compared to published Varian FF and FFF data, suggesting that the percentage surface and build‐up doses as well as the percentage dose difference between FF and FFF beams by our Elekta linac were smaller than those by the Varian linac.

## 1 INTRODUCTION

In radiation therapy, surface and initial build‐up dose is an important measure to take skin toxicity into account.^1^, ^2^ Flattening‐filter‐free (FFF) photon beams have been increasingly used for stereotactic hypofractionated radiotherapy due to their much higher dose rate thereby minimizing the delivery time.^3^ The shorter treatment time may increase the tumor localization accuracy while reducing patient burden. There have been many articles reporting FFF beam characteristics^4^, ^5^, ^6^; however, the number of publications reporting surface and build‐up dose measurement for the FFF beams is limited. Wang et al reported accurate surface and build‐up dose measurement results using a small parallel plate ionization chamber with Varian FFF beams, showing that the build‐up dose resulting from the 6 MV FFF beams was approximately 10% larger than that from 6 MV flattening filter (FF) beams at a depth of 1 mm where the maximum difference was observed.^7^ To the author’s knowledge, no equivalently‐accurate measurement was reported for Elekta FFF beams. The purpose of this study is to report FFF surface dose measurement results with an Elekta linac and compare them to those by corresponding FF beams. Another purpose of this study is to compare the measured Elekta surface and build‐up doses with published Varian linac doses.

## 2 MATERIALS AND METHODS

Using a plane‐parallel advanced Markus chamber, PTW 34045 (PTW, Freiburg, Germany) and a stack of water‐equivalent solid phantom blocks, Tough Water (Kyoto Kagaku, Kyoto, Japan) having thicknesses of 1, 2, 3, 5, 20, 30, 50 mm, the surface and build‐up doses of 6 MV Elekta Synergy (Elekta, Crawley, UK) FF and FFF beams were measured as a function of the phantom depth for field sizes between 2 × 2 and 10 × 10 cm^2^. In an Elekta linac equipped with an Agility multileaf collimator (MLC), a radiation field is defined by MLC leaves and orthogonal jaws.

As an electrometer, Advanced Therapy Dosimeter 35040 (Fluke Biomedical, Everett, U.S.A.) was employed. The parallel plate ionization chamber has the following specifications: a nominal sensitive volume of 0.02 cm^3^ with a depth of 1 mm and a radius of 2.5 mm, a water‐equivalent window thickness of 0.025 mm (protection cap removed), a window area of 20 mm^2^, a protection cap (polymethyl methacrylate, 0.87mm), a plate separation of 1 mm. The small plate separation and sensitive volume along with a thin window thickness allows us to accurately measure an absorbed dose at each depth. The plane‐parallel chamber and the electrometer were calibrated in Association for Nuclear Technology in Medicine (Tokyo, Japan), which is a secondary standard dosimetry laboratory. The density and the uniformity of the water‐equivalent solid phantom blocks were verified by acquiring CT images. The quality assurance of the linac was performed according to the AAPM TG‐142 report.^8^

**Fig. 1:**
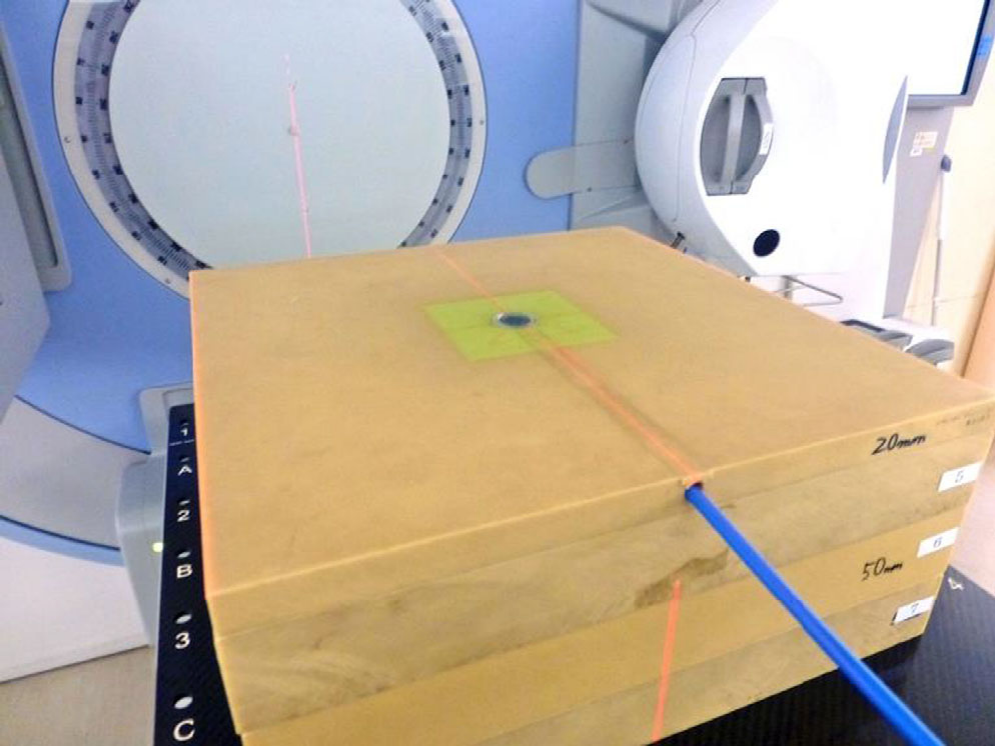
Surface dose measurement setup using a plane‐parallel advanced Markus chamber and a stack of water‐equivalent solid phantom blocks.

Fig. 1 shows the measurement setup where the advanced Markus ionization chamber was embedded into a custom solid water phantom having a thickness of 20 mm. This study was performed using the solid phantom in accordance with the previous study,^7^ where the protection cap of the advanced Markus ionization chamber for the waterproof was removed during the measurement. The source to surface distance was 100 cm. By changing stacked phantom blocks of various thicknesses, depth doses along the beam axis were measured for different field sizes.

A dose of 100 MU was delivered to the phantom with the FF beams having a dose rate of 530 MU/min, whereas a dose of 300 MU was delivered to the phantom with the FFF beams having a dose rate of 1500 MU/min. The measurement was repeated three to five times for averaging purpose.

Percent depth ionization was calculated by normalizing the chamber reading against the reading at a depth of 16 mm where the dose was maximum. Chamber over‐response in the build‐up region as well as polarity effects were not compensated in this calculation similar to a previous paper.^7^ However, the over‐response is considered in the Discussion section when the measured data are compared to published data using a different ionization chamber with a Varian linac.

Cross‐check was performed using radiochromic films (EBT, International Specialty Products, Wayne, U.S.A.), and DosimetryPRO Advantage (Red) Film Digitizer (VIDAR Systems Corporation, Herndon, U.S.A.). A dose of 200 MU with a field size of 10 × 10 cm^2^ was delivered to the phantom with the 6 MV FF and FFF beams. A 7 × 7 pixels median filter was used for denoizing the radiochromic film readings. Absorbed doses on the film were obtained by using the digitized pixel values and a calibration curve. The measurements were repeated five times using data adjacent (‐2 to 2 pixels, pixel size = 0.356 mm) to the beam axis for averaging purpose. Percentage depth doses (PDDs) were calculated by normalizing the doses against the dose at a depth of 16 mm.

## 3 RESULTS

**Fig. 2:**
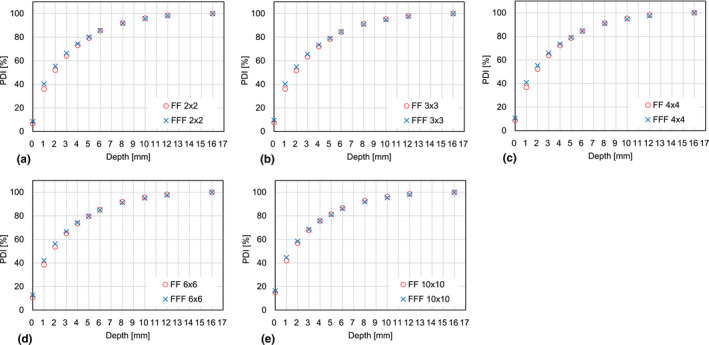
Measured percent depth ionization (PDI) as a function of the phantom depth. Comparisons were made between flattening filter (FF) and FF free (FFF) beams with a photon energy of 6 MV and five different field sizes of (a) 2 × 2, (b) 3 × 3, (c) 4 × 4, (d) 6 × 6, and (e) 10 × 10 cm^2^.

**Fig. 3:**
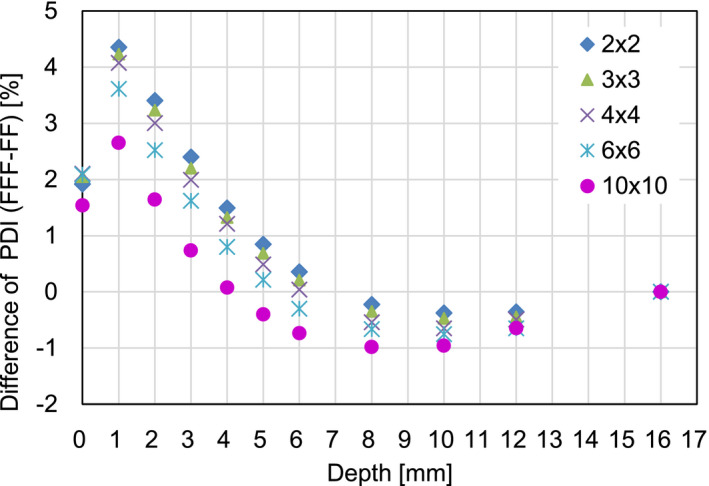
Differences of the percent depth ionizations between 6 MV flattening filter free (FFF) and FF beams as a function of the depth for the five different field sizes.

Table 1 and Fig. 2 show measured percent depth ionizations (PDIs) as a function of the phantom depth. Compared to the differences between the means under the same depths and field sizes, the corresponding standard deviations were relatively small in all cases except. Comparisons were made between FF and FFF with a photon energy of 6 MV and five different field sizes of (a) 2 × 2, (b) 3 × 3, (c) 4 × 4, (d) 6 × 6, and (e)10 × 10 cm^2^. All standard deviations (SDs) of the mean were <0.3 mm. Fig. 3 depicts differences of the PDI between 6 MV FFF and FF beams as a function of the depth for the five different field sizes. It was found that the dose difference between the FF and the FFF beams was relatively small. The maximum difference decreased from 4.4 down to 2.7% as the field size was increased, and the maximum was always observed at a depth of 1 mm, independent of the field size.

**Table 1:** Summary of buildup doses for 6MV flattening filter (FF) and FF free (FFF) beams.

Depth [mm]	2 × 2		3 × 3		4 × 4		6 × 6		10 × 10	
	Mean	SD	Mean	SD	Mean	SD	Mean	SD	Mean	SD
FF: field size (cm)										
0	6.56	0.00	7.48	0.02	8.52	0.01	10.58	0.01	14.86	0.02
1	35.95	0.01	36.01	0.03	36.76	0.02	38.46	0.04	41.99	0.04
2	52.00	0.02	51.62	0.03	52.22	0.04	53.68	0.02	56.73	0.05
3	63.98	0.04	63.36	0.03	63.80	0.02	65.01	0.02	67.66	0.06
4	72.91	0.02	72.07	0.06	72.32	0.06	73.42	0.02	75.63	0.07
5	79.29	0.05	78.37	0.04	78.59	0.03	79.50	0.06	81.45	0.10
6	85.41	0.01	84.40	0.05	84.51	0.03	85.23	0.02	86.78	0.08
8	92.04	0.07	91.30	0.04	91.38	0.08	91.85	0.05	92.90	0.10
10	96.06	0.03	95.35	0.04	95.37	0.04	95.74	0.02	96.39	0.11
12	98.47	0.07	98.07	0.06	98.01	0.05	98.23	0.05	98.57	0.09
16	100.00	0.01	100.00	0.04	100.00	0.05	100.00	0.02	100.00	0.12
FFF: field size (cm)										
0	8.48	0.01	9.53	0.01	10.62	0.02	12.66	0.02	16.40	0.02
1	40.31	0.03	40.24	0.09	40.84	0.06	42.07	0.05	44.65	0.04
2	55.40	0.02	54.86	0.06	55.22	0.14	56.21	0.08	58.37	0.06
3	66.37	0.01	65.56	0.06	65.79	0.10	66.63	0.08	68.40	0.04
4	74.40	0.03	73.40	0.18	73.53	0.15	74.22	0.19	75.71	0.19
5	80.14	0.03	79.06	0.05	79.08	0.12	79.71	0.13	81.05	0.04
6	85.76	0.06	84.61	0.03	84.56	0.13	84.93	0.16	86.04	0.12
8	91.81	0.13	90.95	0.06	90.84	0.24	91.19	0.17	91.92	0.16
10	95.69	0.04	94.88	0.09	94.72	0.24	94.99	0.12	95.43	0.27
12	98.12	0.09	97.63	0.12	97.52	0.15	97.58	0.19	97.92	0.07
16	100.00	0.03	100.00	0.03	100.00	0.20	100.00	0.16	100.00	0.06

**Fig. 4:**
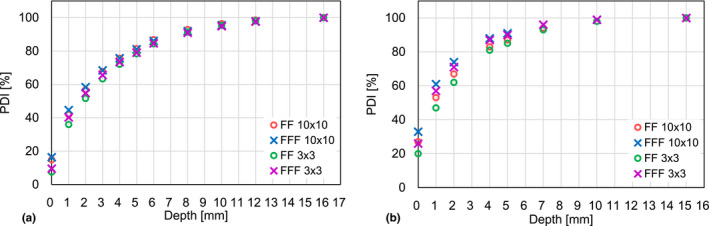
Measured PDI as a function of depth for (a) Elekta and (b) Varian 6 MV flattening filter (FF) and FF free (FFF) beams with different field sizes. Varian data were from a published paper.

Fig. 4 indicates measured PDI as a function of depth for (a) Elekta and (b) Varian 6 MV FF and FFF beams, where the Varian data were taken from a published paper.^7^ It was observed that the percentage surface and build‐up doses by Elekta linac were generally smaller than those by Varian linac.

**Fig. 5:**
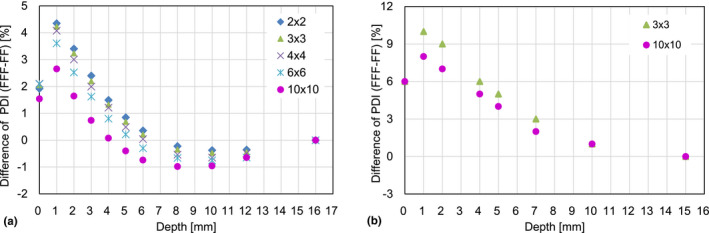
The differences of the PDIs between 6 MV flattening filter (FF) and FF free (FFF) beams as a function of the depth for (a) Elekta and (b) Varian linacs.

Fig. 5 demonstrates the differences of the PDI between 6 MV FF and FFF beams as a function of depth for (a) Elekta and (b) Varian linacs. It was again observed that the PDI differences between FF and FFF beams by Elekta linac were smaller than those by Varian linac.

**Fig. 6:**
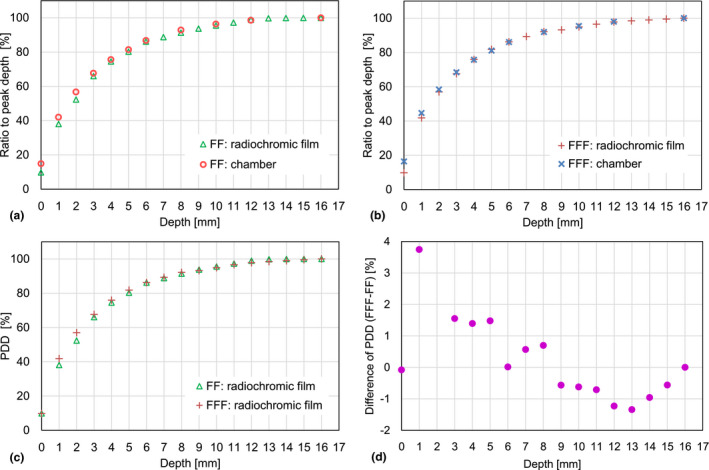
Cross‐check between the radiochromic film and the chamber measurements as a function of depth for (a) flattening filter (FF) and (b) flattening filter free (FFF) beams. (c) Comparison of radiochromic film with FF and FFF beams. (d) Difference of percentage depth dose (PDD) between FF and FFF beams.

Table 2 shows means and SDs of the PDDs using the radiochromic films for the 6 MV FF and FFF beams. The SDs of the PDD by the film were larger than those by the chamber measurements. Fig. 6(a) and 6(b) show cross‐check plots between the radiochromic film and the chamber measurements as a function of depth for the FF and FFF beams. The consistency of the doses measurement was reasonably confirmed. Fig. 6(c) and 6(d) show the differences of the PDD by the film between the FF and FFF beams. Similar to the chamber measurements, the radiochromic film results showed that the FFF dose was larger than the FF dose in the region near the surface, whereas the FF dose was larger than the FFF dose in the region near the peak depth.

**Table 2:** Means and standard deviations (SDs) of the PDDs using the radiochromic films for flattening filter (FF) and FF free (FFF) beams as a function of the depth for 10 × 10 cm^2^.

Depth [mm]	FF		FFF	
	Mean	SD	Mean	SD
0	9.82	0.26	9.74	0.15
1	38.04	0.22	41.79	0.15
2	52.33	0.20	56.91	0.18
3	66.02	0.41	67.57	0.20
4	74.52	0.16	75.91	0.18
5	80.27	0.13	81.75	0.17
6	86.13	0.13	86.15	0.22
7	88.75	0.22	89.31	0.06
8	91.37	0.31	92.07	0.11
9	93.70	0.27	93.14	0.08
10	95.50	0.39	94.88	0.10
11	97.24	0.36	96.53	0.05
12	98.96	0.37	97.73	0.06
13	99.78	0.43	98.44	0.04
14	99.90	0.34	98.94	0.06
15	99.99	0.12	99.43	0.11
16	100.00	0.00	100.00	0.00

## 4 DISCUSSION

Table 1 indicates that the measurement errors for all depths were relatively small compared to the differences between the corresponding means; in other words, the plots in Fig. 2 are reasonably reproducible. The surface and build‐up dose differences between FF and FFF beams for an Elekta linac were relatively small compared to those for Varian linac as shown in Fig. 2 and 4. There are a lot of differences between the two linacs, for example, the head design, and the positions of the collimator jaws and the MLC. The difference of the surface and build‐up doses between the two linacs may be explained by the difference in the number of scattered particles generated inside the linac head as shown in the Fig. 1 of the reference.^1^ In other words, for a given x‐ray field size, Elekta linac provides a smaller solid angle from a measurement point toward the flattening filter and therefore a smaller number of low energy scattered particles reaches the measurement point. In addition, previous studies suggest that the lower position of the Varian MLC may result in higher surface and build‐up doses in comparison to an Elekta machine. Fig. 5 also confirms that the surface and build‐up dose differences between FF and FFF beams for a Varian linac were relatively large compared to those for an Elekta linac. This can be explained by the fact that the Elekta 6 MV FFF beam is energy‐matched to the 6 MV FF beam.^9^ In contrast, it was reported that Varian FFF beam has a lower effective energy than Varian FF beam^9^ thereby possibly increasing surface and build‐up doses compared to the FF beam as shown in Figs. 4(b) and 5(b). High‐precision radiotherapy such as intensity modulated radiotherapy (IMRT) and volumetric modulated arc therapy (VMAT) tends to increase MU. Lower surface and build‐up doses may minimize skin toxicity.

It is also well known that the ionization chamber reading needs to be corrected to compensate the chamber over‐response in the initial build‐up region.^10^ This is required to accurately measure the surface and the build‐up doses using a plane‐parallel ionization chamber. Based on a proposed correction formula,^11^ it was found that the Advanced Markus chamber employed in this study resulted in a PDI correction of 3.4% at the surface while exponentially decreasing by a factor of 1/e at a depth of d_max_/4 (4 mm when d_max_ = 16 mm), meaning that the corrected PDI at the surface and at the depth of 4 mm is 3.4% and 1.3% smaller than the data shown in Fig. 2, respectively. In the published Varian article,^7^ a different ionization chamber, PTW 23342, was employed. In this case, a correction of 4.9% was obtained at the surface, which means that the corrected PDI at the surface is 4.9% smaller than the data shown in the Wang paper with similar exponential decay mentioned above. Table 3 shows the parameters used for calculating the ionization chamber over‐response. The above calculation suggests that the difference of the over‐response between the two chambers is relatively small compared to the difference of the PDIs between Elekta and Varian beams shown in Fig. 4. Although the plane‐parallel chamber in this study has different dimensions from the published Varian article, this study suggested that it is possible to compare the two plane‐parallel chamber readings by correcting over‐responses for both chambers. Our findings are that the surface and build‐up doses were smaller and the dose differences between FF and FFF beams were smaller in the Elekta linac even after correcting the over‐responses.

**Table 3:** Parameters used for chamber over‐response calculation.

	PTW 34045*	PTW23342**
electrode separation (mm)	1	1
sidewall diameter (mm)	9	5.2
wall density (g/cm^3^)	1.17(PMMA)	0.93(PE)

* our study

** reference study

Fig. 6 indicates the consistency between the plane‐parallel ionization chamber and the film measurements for the 6 MV FF and FFF beams. Since the measurement uncertainty in the film dosimetry is generally considered relatively high compared to that of ionization chambers, we only discussed data measured by the plane‐parallel ionization chamber.

Limitation of this study is that only 6 MV beams were considered without polarity correction. Future study includes 10 MV FF and FFF beam comparisons as well as full over‐response and polarity corrections.

## 5 CONCLUSION

Percentage surface and build‐up doses of Elekta 6 MV FF and FFF beams were measured and compared to each other. The data were also compared to published data by Varian beams. It was found that the percentage surface and build‐up doses as well as the percentage dose difference between FF and FFF beams by Elekta linac were considerably smaller than those by Varian linac.

## CONFLICT OF INTEREST

None.
